# Predictors of pneumococcal vaccination among older adults with pneumonia: findings from the Community Acquired Pneumonia Impact Study

**DOI:** 10.1186/1471-2318-10-44

**Published:** 2010-06-30

**Authors:** Paul Krueger, Oona St Amant, Mark Loeb

**Affiliations:** 1 Department of Family and Community Medicine, University of Toronto, Toronto, Ontario, Canada; 2 Arthur Labatt Family School of Nursing, University of Western Ontario, London, Ontario, Canada; 3 Department of Clinical Epidemiology and Biostatistics, McMaster University, Hamilton, Ontario, Canada; 4 Departments of Pathology and Molecular Medicine, McMaster University, Hamilton, Ontario, Canada; 5 Michael DeGroote Institute for Infectious Diseases, McMaster University, Hamilton, Ontario, Canada

## Abstract

**Background:**

The incidence of community-acquired pneumonia (CAP) almost triples for older adults aged 65 years or older. In Canada, CAP is a leading cause of hospital admissions and mortality. Although CAP is very prevalent, complications due to CAP may be reduced with the pneumococcal polysaccharide vaccine (PPV). The purpose of this study was to identify predictors of pneumococcal vaccination among community-dwelling older adults with clinically diagnosed CAP.

**Methods:**

A telephone survey was used to collect detailed information from adults aged 60 years and older with clinically diagnosed CAP. This was a community wide study with participants being recruited from all radiology clinics in one Ontario community.

**Results:**

The most important predictors of pneumococcal vaccination among older adults included: getting an influenza vaccine within the past year (OR 14.5, 95% CI 4.27 to 49.0); at least weekly contact with a friend (OR 3.97, 95% CI 1.71 to 9.24); having one or more co-morbidities/chronic conditions (OR 3.64, 95% CI 1.60 to 8.28); being 70 years of age or older (OR 2.56, 95% CI 1.21 to 5.40); having health problems that limited physical activities (OR 5.37, 95% CI 1.49 to 19.3); having little or no bodily pain (OR 2.90, 95% CI 1.25 to 6.73); and reporting having spiritual values or religious faith (OR 3.47, 95% CI 1.03 to 11.67).

**Conclusions:**

A wide range of factors, including demographic, co-morbidity, quality of life, social support and lifestyle were found to be associated with pneumococcal vaccination status among older adults with clinically diagnosed CAP. The findings from this study could inform future pneumococcal immunization strategies by identifying individuals who are least likely to receive the PPV.

## Background

Community-acquired pneumonia (CAP) refers to pneumonia acquired outside of hospitals or extended-care facilities [1]. In Canada, CAP poses a considerable threat to the health of older adults and the incidence of CAP almost triples among those aged 65 years or older [2]. With the pending demographic influx of seniors, the prevalence of CAP is expected to substantially increase, resulting in a greater burden for older adults, their caregivers and the health care system [3]. CAP is a leading cause of hospital admissions and mortality in Canada [2,4,5] and with increasing age, there is a corresponding increase in morbidity and loss of independence for older adults [6,7]. Furthermore, case fatality rates of pneumonia with invasive pneumococcal disease increase sharply from 20% (for person 65 years or older) to 40% (for persons 85 years or older) [8]. Seniors with cardiopulmonary disease, poor functional status (such as limitations with activities of daily living), weight loss or recent changes in weight are at increased risk for CAP [9].

The most common causative pathogen worldwide of CAP is *Streptococcus Pneumoniae*, accounting for approximately 30-50% of all cases [10]. The pneumococcal polysaccharide vaccine (PPV-23) immunizes against 23 strains of the pneumococcus bacteria [11]. PPV is 50-80% effective in the prevention of invasive pneumococcal disease among immunocompetent patients [11-13]. While the efficacy of the pneumococcal vaccine in preventing pneumonia remains inconclusive, [12,14-16] there is evidence that administration of the vaccine can play a critical role in reducing the severity of the disease among older adults [15].

Despite recommendations for uptake of PPV in seniors and a target goal of 80% immunization rate among adults aged 65 years or older by the year 2010 in Canada, [11-13,17] immunization rates remain low with only 42% of Canadians over 65 years of age reported being vaccinated [13,17,18]. In order to further understand PPV uptake in Canada, where the vaccine is publically funded, we explored the predictors for pneumococcal vaccination among community-dwelling older adults with clinically diagnosed CAP.

## Methods

The Community Acquired Pneumonia Impact Study (CAPIS) was a mixed methods, community-based study designed to assess the impact of CAP on older adults and their family caregivers. This manuscript reports the findings from the quantitative data, specifically focusing on predictors of pneumococcal vaccination among older adults having a clinical diagnosis of CAP. Other qualitative and quantitative findings are reported elsewhere [19,20].

### Setting

This study was conducted in Brant County, Ontario, a mix of urban and rural settings which includes the city of Brantford and the amalgamated County of Brant (comprised of eight towns and villages). The population of Brant County at the time of data collection was 118,485 with 14% of the population aged 65 years and older. Brant County was selected for this community-based study because of its moderate size and population demographics. The population of Brant County is predominantly English-speaking, with 86% reporting English as a first language. There were two major community hospitals, eight radiology centers and approximately 80 family physicians at the time of the study.

### Recruitment

Study participants were recruited over a 15 month period at the eight x-ray facilities in Brant County. Eligibility criteria included being clinically diagnosed with CAP by a family or emergency room physician, being 60 years of age or older, living in the community (Brant County), presenting for a chest x-ray at one of the community or hospital radiology centres, speaking English, and obtaining informed consent. Exclusion criteria included: cognitive impairment and having hospital or nursing home acquired pneumonia. In order to preserve the health of the participants and prevent participant-burden, x-ray technicians were trained to recruit participants at the time of their x-ray and a trained interviewer telephoned the participants four weeks later. Ethics approval was obtained from McMaster University and the Brant Community Health Care System.

### Data Collection

The interviewer collected detailed information including: demographic characteristics (gender, age, marital status, living arrangements (i.e. number, ages and relationships of people living in the household; owning or renting; type of dwelling), cultural background (i.e. the ethnic or cultural group most identified as representing their heritage), level of education (categories from none to a university graduate degree), household income (total household income before taxes and deductions in $20 k increments), perceived level of social status (as measured by the MacArthur Scale of Subjective Social Status), employment history (whether currently employed; main occupation when employed); co-morbidities (e.g. allergies, asthma, chronic bronchitis, diabetes, emphysema, heart disease, cancer and liver disease); lifestyle (e.g. immunizations (if ever received the influenza and pneumonia vaccines; and the timing either < 1 year ago; 1 to 2 years ago; more than 2 years ago), having a family physician, smoking status, exposure to second hand smoke, alcohol consumption (ever in the past 12 months), ownership of pets, nutrition (frequency skipping meals, number of servings of fruits, vegetables, milk products, meal replacements/supplements; difficulty chewing or swallowing; self perceived appetite), spiritual values (rating of how much spiritual values or religious faith plays a role in their life), overall happiness (rating from very unhappy to very happy); quality of life (using the Short-Form-8 Health Survey (SF-8) to collect information on overall health, activity limitation because of health problems, difficulty doing usual daily activities because of physical health, amount of bodily pain, level of energy, limitations of social activities and activities due to personal or emotional problems); functional status (measured using the 10-item Modified Barthel Index which includes grooming, dressing, feeding oneself, transferring from one's bed to a chair, bathing, toilet use, bladder control, bowel control, mobility, climbing stairs); instrumental activities of daily living scale (measured using the 8-item Instrumental Activities of Daily Living Scale developed Lawton which includes items on meal preparation, mobility beyond short distances, shopping, phone calling, doing laundry, doing household work or handymen work, taking one's medication and money managing); and social support (numbers and types of family relatives, friends, distance to these contacts, frequency of contact, involvement in social and religious networks). Data collection ended March 2004.

### Data analysis

The dependent variable was self reported pneumococcal vaccination status prior to receiving a clinical diagnosis of CAP. The dichotomous outcome variable was created based on participant responses, namely, "ever vaccinated" and "never vaccinated". Based on the literature and clinical experience, two investigators (PK and ML) reviewed the questionnaire for potential predictors to include in the analysis. The potential predictor variables included the above listed demographic characteristics, co-morbidities, lifestyle, quality of life, functional status, instrumental activities of daily living, and social support variables. Data from the telephone interviews were entered into and analyzed using SPSS 17.0 (SPSS Inc., Chicago IL). Descriptive statistics were computed for all variables, including frequency counts, and percentages for categorical variables, or means and standard deviations for continuous variables. For categorical variables, we used the chi-squared test, or when appropriate, Fisher's exact test to determine the significance of potential predictor variables. In addition, unadjusted odd ratios (ORs) and 95% confidence intervals (CIs) are reported for each potential predictor of pneumococcal vaccination. T-tests were used to compare continuous variables between the vaccinated and non-vaccinated patients.

A logistic regression analysis was used to identify the best predictors of pneumococcal vaccination status from those variables which had a statistically significant association in the above bivariate analyses or were considered by the investigators to be theoretically significant. A forward selection process was used whereby non-significant variables were removed from the model one at a time. The parameter estimates were reviewed at each step to assess whether the eliminated variable should be kept in the model to control for confounding. Adjusted ORs and corresponding 95% CIs are reported for each variable in the final logistic regression model. The goodness of fit of the logistic regression model was assessed using rho-square statistic [21]. A rho-square value between 0.20 and 0.40 suggests a very good fit of the model. The Cox and Snell (R^2^) and Nagelkerke (R^2^) statistics are also reported as estimates of the proportion of variance explained by the final model. A probability level of < .05 was used to determine statistical significance.

## Results

### Sample Characteristics

Forty-four potentially eligible patients refused consent to participate and therefore no information was available for these individuals. Of those who initially agreed to participate, 86% completed the telephone interview. The reason for declining was that the patients simply did not want to participate. Of the 195 participants, 95 had x-ray confirmed CAP, and 185 reported on their pneumococcal vaccination status. Among these participants: 62% were female; 66% were aged 70 years or older (mean 72.7 years, standard deviation 6.7); 62% were married or common-law; 71% lived with others; 76% owned their homes; 54% completed high school; 68% earned a household income of $20,000 or more; and 94% had children.

### Bivariate Analysis

A total of 58 variables were identified *a priori *from the telephone interview data as potential predictors of pneumococcal vaccination status. Of these 58 variables, 19 were included in the logistic regression analysis based on either their statistical or theoretical significance (Table 1).

**Table 1:** Potential predictors of pneumococcal vaccination among older adults (aged 60 years of age and older) with clinically diagnosed1 community acquired pneumonia (n = 185).

	Pneumococcal Vaccination Status^2^				
Potential Predictor Variables	Ever Vaccinated (n = 102)	Never Vaccinated (n = 83)	P-value^3^	Odds Ratio^4^	95% CI
Demographic Variables					

Participant's age (n = 183):					
60 to 69	27 (42.9)	36 (57.1)		1.00	-
70 to 90	75 (62.5)	45 (37.5)	0.011	2.22	(1.19, 4.14)

Co-morbidities					

Reported health condition (asthma) (n = 185):					
No	84 (51.9)	78 (48.1)		1.00	-
Yes	18 (78.3)	5 (21.7)	0.017	3.34	(1.18, 9.44)

Reported health condition (chronic bronchitis) (n = 185):					
No	77 (52.0)	71 (48.0)		1.00	-
Yes	25 (67.6)	12 (32.4)	0.089	1.92	(0.90, 4.11)

Reported health condition (diabetes) (n = 185):					
No	85 (52.8)	76 (47.2)		1.00	-
Yes	17 (70.8)	7 (29.2)	0.097	2.17	(0.85, 5.52)

Reported health condition (emphysema) (n = 185):					
No	90 (52.3)	82 (47.7)		1.00	-
Yes	12 (92.3)	1 (7.7)	0.007^5^	10.93	(1.39, 85.9)

Reported health condition (congestive heart failure) (n = 185):					
No	93 (53.8)	80 (46.2)		1.00	-
Yes	9 (75.0)	3 (25.0)	0.152	2.58	(0.68, 9.86)

None of the above health conditions (n = 185):					
Had none	21 (41.2)	30 (58.8)		1.00	-
Had at least one	81 (60.4)	53 (39.6)	0.019	2.18	(1.13, 4.21)

Lifestyle					

Most recent influenza vaccine (n = 185):					
One year or ore/never	4 (15.4)	22 (84.6)		1.00	-
Less than one year ago	98 (61.6)	61 (38.4)	<0.001	8.84	(2.91, 26.9)

Before illness, how often did the participant skip meals (n = 185):					
Sometimes/often/daily	13 (36.1)	23 (63.9)		1.00	-
Never/rarely	89 (59.7)	60 (40.3)	0.011	2.62	(1.23, 5.58)

How much does spiritual values or religious faith play a role in life (n = 184):					
None	7 (36.8)	12 (63.2)		1.00	-
Great deal to little	95 (57.6)	70 (42.4)	0.085	2.33	(0.87, 6.21)

Quality of life (SF-8) ratings before illness					

Overall rating of health (n = 185):					
Excel/very good/good	84 (53.2)	74 (46.8)		1.00	-
Fair/poor/very poor	18 (66.7)	9 (33.3)	0.192	1.76	(0.75, 4.16)

Before illness, how much did the participant's health problems limit your usual activities (n = 185):					
Somewhat to not at all	82 (51.3)	78 (48.8)		1.00	-
Quite a lot/couldn't do	20 (80.0)	2 (20.0)	0.007	3.81	(1.36, 10.6)

How much does the participant have difficulty doing usual daily activities because of physical health (n = 185):					
Somewhat to not at all	88 (53.0)	78 (47.0)		1.00	-
Quite a lot/couldn't do	14 (73.7)	5 (26.3)	0.086	2.48	(0.86, 7.20)

How much bodily pain does the participant have (n = 184):					
Mod/severe/very severe	54 (65.1)	25 (24.8)		1.00	-
None/very mild/mild	76 (75.2)	29 (34.9)	0.131	1.63	(0.86, 3.10)

How much energy does the participant have (n = 185):					
Very much/a lot/some	91 (52.6)	82 (47.4)		1.00	-
A little/none	11 (91.7)	1 (8.3)	0.013^5^	9.91	(1.25, 78.5)

Functional Status (before illness)					

Barthel 10-item ADL score (n = 184):					
20	78 (52.0)	72 (48.0)		1.00	-
9 to 19^6^	23 (67.6)	11 (32.4)	0.098	1.93	(0.88, 4.24)

Social support					

Does the participant have sister(s) and/or brother(s) (n = 184):					
Yes	72 (52.2)	66 (47.8)		1.00	-
No	30 (65.2)	16 (34.8)	0.123	1.72	(0.86, 3.44)

Distance to the participant's nearest sibling (n = 122):					
Within Brant	22 (40.7)	32 (59.3)		1.00	-
Outside Brant	39 (57.4)	29 (42.6)	0.068	1.96	(0.95, 4.04)

How often in the last 6 months has the participant seen or spoken with above relatives on the phone (n = 184):					
Less than once/week	4 (30.8)	9 (69.2)		1.00	-
At least once/week	98 (57.3)	73 (42.7)	0.063	3.02	(0.90, 10.2)

How often in the last 6 months participant had a chat or did something with a friend (n = 184):					
Less than once/week	16 (37.2)	27 (62.8)		1.00	-
At least once/week	86 (61.0)	55 (39.0)	0.006	2.64	(1.30, 5.34)

^1^All participants sent for chest x-ray by physicians to rule out/confirm clinically suspected CAP.

^2^Self reported.

^3^Chi-square test.

^4^Unadjusted odds ratios.

^5^Fisher exact test.

^6^One participant had a score of 9, all others in this category scored either 18 or 19.

### Multivariable Analysis

The 19 variables shown in Table 1 were entered into a logistic regression analysis. The final logistic regression model included seven variables (Table 2): getting an influenza vaccine within the past year (OR 14.5, 95% CI 4.27 to 49.0); at least weekly contact with a friend (OR 3.97, 95% CI 1.71 to 9.24); having one or more co-morbidities/chronic conditions (OR 3.64, 95% CI 1.60 to 8.28); being 70 years of age or older (OR 2.56, 95% CI 1.21 to 5.40); having health problems that limited physical activities (OR 5.37, 95% CI 1.49 to 19.3); having little or no bodily pain (OR 2.90, 95% CI 1.25 to 6.73); and reporting having spiritual values or religious faith (OR 3.47, 95% CI 1.03 to 11.67). The final logistic regression model statistics are reported in Table 2.

**Table 2:** Logistic regression of the most important predictors of pneumococcal vaccination among older adults with clinically diagnosed community-acquired pneumonia and sent for confirmatory x-rays (n = 181^1^).

Predictors of Pneumococcal Vaccination	Adjusted Odds Ratio^2^	95% Confidence Interval
Most recent influenza vaccine:		
One year or more/never	1.00	-
Less than one year ago	14.46	(4.27, 49.0)

How often in the last 6 months participant had a chat or did something with a friend:		
Less than once/week	1.00	-
At least once/week	3.97	(1.71, 9.24)

Reported having health conditions in list^3^:		
Reported none	1.00	-
Reported at least one	3.64	(1.60, 8.28)

Participant's age:		
60 to 69	1.00	-
70 to 90	2.56	(1.21, 5.40)

Before illness, how much did your health problems limit your usual activities^4^:		
Somewhat, very little, not at all	1.00	-
Quite a lot, could not do physical activities	5.37	(1.49, 19.31)

Amount of bodily pain^4^:		
Mod/severe/very severe	1.00	-
None/very mild/mild	2.90	(1.25, 6.73)

How much spiritual values or religious faith plays a role in life:		
None	1.00	-
Great deal to little	3.47	(1.03, 11.67)

***Final Logistic Regression Model Statistics***:

Rho-square = 0.25 (pseudo R^2^, values between 0.2 and 0.4 suggest a very good fit)

Cox & Snell R-square = .292; Nagelkerke R-square = .391 (i.e. between 29.2% and 39.1% of variance is explained by this model)

Hosmer and Lemeshow Goodness-of-Fit test = 0.948 (values greater than 0.25 indicate good fit)

74.6% correctly classified

^1^Four of the 185 (2.2%) participants had missing values for one or more of the variables included in the final model.

^2^Odds ratios for categorical variables represent comparisons with the referent group (OR = 1.00) after adjustment for all other variables in the model. An odds ratio greater than one indicates increased likelihood for pneumococcal vaccination. For example, participants aged 70 and older were 2.56 times more likely to report receiving the pneumococcal vaccination than participants 60 to 69 years of age (after adjusting for all other variables in the model).

^3^List included: food allergies, other allergies, asthma, chronic bronchitis, diabetes, emphysema, heart disease, congestive heart failure, cancer, liver disease, kidney disease, ever received a transplant, taking immunosuppressant drugs.

^4^Question from SF-8.

## Discussion

From our dataset, we identified seven important predictors of pneumococcal vaccination. Those who reported getting an influenza vaccine within the past year were more likely to report having received the pneumococcal vaccine than those who had not. Our results differ from those of Al-Sukhni et al. who did not find a statistically significant association between regular annual receipt of the influenza vaccine and the likelihood of pneumococcal vaccination (OR 0.90; 95% CI 0.45-1.79; p-value = 0.75) [13]. The authors, however, reported that most participants (59%, 92/156) reported receiving the PPV at the same time as the influenza vaccine [13]. The timing of the vaccine (and therefore the opportunity to receive PPV) may be related to the influenza vaccine as a predictor for pneumococcal vaccination.

Older adults who reported chatting or doing something with a friend at least once/week were more likely to report having received the pneumococcal vaccine. This finding is in keeping with what may be reasonably expected. A study by Madhavan et al. assessed predictors of influenza and pneumonia vaccination among rural senior adults in the United Kingdom. The authors found that knowing someone with pneumonia was the strongest predictor for the pneumonia vaccination in rural senior adults (p = 0.007) [22]. Older adults tend to talk about their health and a discussion about influenza and or pneumonia could prompt them to make an immunization appointment with their family physician.

Older adults with one or more co-morbidities were also more likely to report having received the pneumococcal vaccine. A likely explanation for co-morbidities as a predictor for pneumococcal vaccination is that persons with chronic conditions are more likely to access health care services more frequently, allowing for more opportunities to engage with health care practitioners. The evidence related to the role of practitioners and pneumococcal vaccination rates, however, is conflicting. Stehr-Green et al. identified a recommendation by a health care provider as the most important predictor of PPV immunization among older adults [23]. In a study based in the same region as this study (Brantford, Ontario) Krueger et al. found that over half of family and ER physicians surveyed reported CAP to be a very important health concern for their practices [24]. In contrast, however, a study examining the impact of public vaccination programs in Ontario found that more than 90% of unvaccinated respondents reported seeing a physician at least once in the previous year, indicating a missed opportunity for vaccination [13]. The authors suggest that this missed opportunity may be related to physicians' on-going uncertainty about the effectiveness of the vaccine.

Older study participants were more likely to report having received the pneumococcal vaccine. This finding is also in keeping with what may be reasonably expected. Since the highest incidence of pneumonia occurs among people > 85 years of age (81 cases per 100,000) in Canada [14], it is more likely that these older patients would be targeted for immunizations by family physicians. Similarly, older adults who identified that their health problems (prior to their bout of pneumonia) limited their usual activities a lot, or prevented them from doing physical activities, were more likely to report having received the pneumococcal vaccine. Again, this could be due to a greater likelihood or frequency of contact with their family physicians.

The finding that older adults with mild to no bodily pain are more likely to have received the pneumococcal vaccine than those with more severe pain may be related to their ability to access health care services. Although few studies have examined the relationship between bodily pain and vaccination status, a study by Groenwold et al. identified bodily pain as a potential unmeasured confounder for immunizations, specifically using the influenza vaccine as an example [25]. The authors found bodily pain to be inversely related to vaccination status. Further research is needed to understand the relationship between bodily pain and the likelihood of immunization [25]. Although somewhat speculative, those with less pain may have less difficulty accessing their family physician or immunization clinic.

The finding that older adults who reported having spiritual values or religious faith was an important predictor of pneumococcal vaccination is interesting. Again, although speculative, this finding could be related to social networking. Those who go to church or attend religious outings may be advised to get their immunizations to avoid illness, or have greater opportunity for talking about immunizations than those who do not have this type of social networking. While the relationship between spiritual values and/or religion and vaccination status has not been explored in-depth in the literature [26], some studies have demonstrated a positive relationship between religion and health promoting behaviors such as healthy eating habit [27].

Strengths of this study include it being a community-based study that attempted to recruit all older adults who were sent for a chest x-ray to confirm/rule out CAP. In addition, we had a comprehensive data set that allowed us to explore the association between a wide range of demographic, health, lifestyle, quality of life, functional status, and social support variables and whether or not community dwelling older adults received the pneumococcal vaccine. There are several potential limitations of this study. The first is that we only recruited older adults who went for chest x-rays. We therefore missed those who were treated for CAP by their physicians but were not sent for chest x-rays or who were sent but did not go. Self-reported immunization status is another potential limitation. The literature would suggest that the sensitivity of self reported pneumococcal vaccination status is very good but there is more variability with reported specificity. However, one potential reason for the variability in specificity is the validity of the source of the comparison data (i.e. medical charts). This is particularly important in Ontario where a relatively high percentage of the population are without a family physician and where older adults have easy access to community immunization clinics outside family physician practices (notices regarding immunization would not be sent to family physicians). The inaccuracy of using medical charts could therefore account for some of the variability in specificity noted in the literature. Sample size was also a limitation of this study, resulting in large confidence intervals. Given the large number of potential predictor variables and the relatively small sample size, another limitation is the chance for Type I error. In defense of this, however, we restricted our analyses to only include meaningful variables that were chosen *a priori *and our multivariate modeling fulfilled the requirement (1 variable for 10 outcome events) for having reliable parameter estimates. Since this study was done in only one relatively homogeneous community, the generalizability of the findings is another potential limitation. Although we expect the accuracy of the information collected from study participants to be very good, based on the use of reliable and valid instruments, some degree of random error should be expected in studies that collect self reported data retrospectively. However, we don't suspect that recall bias is a weakness of this study. And finally, our definition of CAP was clinically diagnosed CAP versus x-ray confirmed CAP. The decision to use clinically diagnosed CAP versus x-ray confirmed CAP was based on there being no important differences in the characteristics or outcomes of those clinically diagnosed versus those with a positive chest x-ray; the fact that a large percentage of physicians do not send their patients for chest x-rays; and to increase the sample size for this analysis.

## Conclusions

In conclusion, this study identified a wide range of factors, including demographic (age), co-morbidity (having at least one health condition; amount of bodily pain), quality of life (the extent that health problems limited usual activities), social support (frequency chatting or doing something with a friend) and lifestyle (recent influenza immunization; and the amount that spiritual values or religious faith played a role in life) to be associated with pneumococcal vaccination status among older adults with clinically diagnosed CAP. CAP is a relatively common infection among community-dwelling elderly. Although there are identified co-morbidity risk factors for CAP, such as chronic lung disease, one of the most important is age with the "older" elderly being at highest risk. Because the risk of invasive pneumococcal disease increases in this group, for which there is excellent evidence that the vaccine is effective, from a health policy perspective this is indeed the group that should be targeted for pneumococcal immunization. The findings of this study, by helping to delineate the likelihood of receiving the vaccine, identify factors that need to be considered when targeting vaccine to the "low-uptake" elderly. Therefore, the findings from this study could inform future pneumococcal immunization strategies in Canada by identifying those individuals who are least likely to receive the PPV.

## Competing interests

The authors declare that they have no competing interests.

## Authors' contributions

PK had a major role in the conception and design of the study, supervised all aspects of the study's implementation, had a major role in the data analysis and interpretation, contributed to the writing of the manuscript, and provided editorial comments. OS had a role in the analysis and interpretation of the data, writing and revision of the manuscript. ML contributed to the conception of the study design, participated in data analysis and interpretation, contributed to the writing of the manuscript and provided editorial comments. All authors read and approved the final manuscript.

## Pre-publication history

The pre-publication history for this paper can be accessed here:

http://www.biomedcentral.com/1471-2318/10/44/prepub
